# M2 Macrophages Promote HCC Cells Invasion and Migration via miR-149-5p/MMP9 Signaling

**DOI:** 10.7150/jca.35444

**Published:** 2020-01-01

**Authors:** Guodong Liu, Lei Yin, Xiwu Ouyang, Ke Zeng, Yao Xiao, Yixiong Li

**Affiliations:** 1 Department of Biliary and Pancreatic Surgery, Xiangya Hospital, Central South University, Changsha, 410008, China.; 2 Department of Urology, Shanghai Tenth People's Hospital, School of Medicine in Tongji University, Shanghai, China.; 3 Department of Liver Surgery, Xiangya Hospital, Central South University, Changsha, 410008, China.; 4 CITIC Xiangya Reproductive and Genetic Specialist Hospital, Changsha, 410008, China.; 5 Department of Hepatobiliary and Pancreatic Surgery, Xiangya Hospital, Central South University, Changsha, 410008, China.

**Keywords:** Liver cancer, MiRNA-149-5p, MMP9, Invasion, Migration

## Abstract

The roles of M2 macrophages on promoting tumor progression and chemotherapy resistance have been well studied in many cancers, such as pancreatic cancer, kidney cancer and so on, but its linkage to HCC cells still remains unclear. Here we found that M2 macrophages could alter miR-149-5p to increase MMP9 expression in HCC cells and mechanism dissection revealed that miR-149-5p might directly target the 3'UTR of MMP9-mRNA to suppress its translation. The in vivo orthotopic xenografts mouse model with oemiR-149-5p also validated in vitro data. Together, these findings suggest that M2 macrophages may through altering the miR-149-5p to promote HCC progression and targeting the M2 macrophages/miR149-5P/MMP9 signaling may help in the development of the novel therapies to better suppress the HCC progression.

## Introduction

Hepatocellular carcinoma (HCC) is the fifth most frequently diagnosed and the second cause of cancer death in the word 1. HCC is one of the most lethal tumors with high recurrence and metastatic rate, which made it one of the poorest-prognosis cancers across the globe. In 2018, 42,220 cases of liver and intra hepatic bile duct cancer were newly diagnosed in the US, and the incidence has almost increased by twice in the past thirty years 2. For advanced HCC patients, sorafenib seems to be one of most useful targeted drugs, but its function is limited by many factors, and new therapies are eagerly yet to be developed.

Tumor microenvironment is very important for the growth, progression and metastasis of tumors, among which, inflammation is one of the most important factors 3-4. Macrophage is one of the most common immune related cells in tumor microenvironment, and its functions to tumors have already been well documented in many cancers, such as pancreatic cancers 5-6, prostate cancers 7-8, lung cancers 9 and so on. Macrophages can be differentiated into classically (M1) and alternatively (M2) activated polar cells based on different environment situations. Usually, M1 macrophages can function as pro-inflammatory effect by expressing nitric oxide synthase (iNOS), whereas M2 macrophages can express anti-inflammatory cytokines, such as IL-10, to promote tumor progression and metastasis. More and more studies have showed that tumor-associated macrophages trend to M2 macrophages, can make tumor cells more invasive and aggressive 10-11.

In this study, we demonstrated that M2 macrophages can alter the expression of miR-149-5p to promote the invasion and migration of HCC by increasing MMP9 pathway, thus we delineated a novel signaling between M2 macrophages and HCC progression and also provide a new possibility for the novel therapies to better suppress the HCC progression.

## Material and Methods

### Cell culture

HCC SK-HEP-1, HEK 293T and human acute monocytic leukemia cell line THP-1 were purchased from the American Type Culture Collection (ATCC). HA22T cell line (BCRC No. 60168) was gifted from Prof. Yuh-Shan Jou, Academia Sinica, Taiwan. HA22T, SK-HEP-1 and HEK 293T cell lines were cultured in DMEM (Invitrogen) with 10% Fetal Bovine Serum, 1% penicillin/streptomycin and 1% glutamine. THP-1 cell line was cultured in RPMI1640, 100 ng/ml Phorbol 12-myristate 13-acetate (PMA, Sigma) was used for 48 hours to induce THP-1 differentiation into macrophages. To induce M2 polarized phenotype, 20 ng/ml of IL-4(Invitrogen) and IL-13 (R&D Systems) were used for 48 hours. All cell lines were cultured in a 5% (v/v) CO2 humidified incubator at 37 °C.

### Lentivirus packaging

The pLKO.1-shMMP9^#1^, pLKO.1-shMMP9^#2^, pWPI-MMP9, pLKO.1-miR-149-5p, pLKO.1-miR-483-3p, pLKO.1-miR-6734-3p plasmids were transfected into 293T cells and cultured in virus room, the lentivirus soup was collected after 48 hours and used immediately or frozen in -80 °C for later use.

The plasmids sequences used were as follows:

pLKO.1-shMMP9^#1^ sequence,

*Forward:* 5'-CCGGCCACAACATCACCTATTGGATGGATCCATCCAATAGGTGATGTTGTGGTTTTTG-3',

*Reverse:* 5'-AATTCAAAAACCACAACATCACCTATTGGATGGATCCATCCAATAGGTGATGTTGTGG -3';

pLKO.1-shMMP9^#2^ sequence,

*Forward:* 5'-CCGGCAGTTTCCATTCATCTTCCAAGGATCCTTGGAAGATGAATGGAAACTGTTTTTG -3',

*Reverse:* 5'-AATTCAAAAAAGTTTCCATTCATCTTCCAAGGATCCTTGGAAGATGAATGGAAACTG -3';

pLKO.1-miR-149-5p sequence,

*Forward:* 5'-CCGGTCTGGCTCCGTGTCTTCACTCCCTTGGATCCGGGGAGTGAAGACACGGAGCCAGA TTTTTG-3',

*Reverse:* 5'-AATTCAAAAATCTGGCTCCGTGTCTTCACTCCCCGGATCCAAGGGAGTGAAGACACGGAGCCAGA-3';

pLKO.1-miR-483-3p sequence,

*Forward:* 5'-CCGGTCACTCCTCTCCTCCCGTCTTTTGGATCCGAAGACGGGAGGAGAGGAGTGA TTTTTG-3',

*Reverse:* 5'-AATTCAAAAATCACTCCTCTCCTCCCGTCTTCGGATCCAAAAGACGGGAGGAGAGGAGTGA-3';

pLKO.1-miR-6734-3p sequence,

*Forward:* 5'-CCGGCCCTTCCCTCACTCTTCTCTCAGTTGGATCCGCTGAGAGAAGAGTGAGGGAAGGG TTTTTG-3',

*Reverse:* 5'-AATTCAAAAACCCTTCCCTCACTCTTCTCTCAGCGGATCCAACTGAGAGAAGAGTGAGGGAAGGG-3'.

### RNA extraction and qRT-PCR analysis

Trizol reagent (Invitrogen, Grand Island, NY) was used to extract total RNA, and 1-2ug of the total RNA was used to reverse transcribe into cDNA by using Superscript III transcriptase (Invitrogen, Grand Island, NY). Relative RNA expression was conducted by Quantitative real-time PCR (qRT-PCR) assay by using a Bio-Rad CFX96 system with SYBR green reagent and the results were normalized by GAPDH.

The primer sequences used were as follows:

CD206 primer,

*Forward:* 5'-GGGTTGCTATCACTCTCTATGC-3',

*Reverse:* 5'-TTTCTTGTCTGTTGCCGTAGTT-3';

CD163 primer,

*Forward*: 5'-TTTGTCAACTTGAGTCCCTTCAC-3',

*Reverse:* 5'-TCCCGCTACACTTGTTTTCAC-3';

Arginase-1 primer,

*Forward:* 5'-GGTTTTTGTTGTTGCGGTGTTC-3',

*Reverse:* 5'-CTGGGATACTGATGGTGGGATGT-3';

TGF β primer,

*Forward:* 5'-CAATTCCTGGCGATACCTCAG-3',

*Reverse:* 5'-GCACAACTCCGGTGACATCAA-3';

IL-10 primer,

*Forward:* 5'-GACTTTAAGGGTTACCTGGGTTG-3',

*Reverse:* 5'-TCACATGCGCCTTGATGTCTG-3';

MMP9 primer,

*Forward:* 5'- TGTACCGCTATGGTTACACTCG-3',

*Reverse:* 5'- GGCAGGGACAGTTGCTTCT-3';

GAPDH primer,

*Forward:* 5'- TGTGGGCATCAATGGATTTGG-3',

*Reverse:* 5'- ACACCATGTATTCCGGGTCAAT-3';

hsa-miR-1224-3p primer: CCCCACCTCCTCTCTCCTCAG;

hsa-miR-149-5p primer: TCTGGCTCCGTGTCTTCACTCCC;

hsa-miR-154-5p primer: TAGGTTATCCGTGTTGCCTTCG;

hsa-miR-183-5p primer: TATGGCACTGGTAGAATTCACT;

hsa-miR-204-5p primer: TTCCCTTTGTCATCCTATGCCT;

hsa-miR-211-5p primer: TTCCCTTTGTCATCCTTCGCCT;

hsa-miR-2355-5p primer: ATCCCCAGATACAATGGACAA;

hsa-miR-483-3p primer: TCACTCCTCTCCTCCCGTCTT;

hsa-miR-491-5p primer: AGTGGGGAACCCTTCCATGAGG;

hsa-miR-6734-3p primer: CCCTTCCCTCACTCTTCTCTCAG.

### Western blot analysis

RIPA lysate was used to lyse the collected cells, and 30ug protein was electrophoresed in 10% SDS/PAGE gel and then transferred onto PVDF membranes. After that, specific primary antibodies were used to incubate the relative bands for overnight in a 4℃ room and then incubated with the secondary antibodies for 1-2 hours and visualized with ECL system (Thermo Fisher Scientific, Rochester, NY). GAPDH (6c5), MMP-9(2C3), c-Myc(6A10), ERK1(E-12), IGF-I(AT6F8), MAO-A(G-10), MMP-1(3B6) and VEGF(C1) antibodies were purchased from Santa Cruz Biotechnology.

### Cell invasion assay

Cell invasion assay was performed by using 8 μm transwell chamber (Corning Life Science) in 24-well plates. 5×10^4^ cells/well were seeded into upper chamber coated with diluted Matrigel (1:20 dilution, 100 μl/well; BD Biosciences) with serum-free medium and 750μL media with 10% FBS /well was added into lower chambers for incubation for 24 hours. The invaded cells were fixed by methanol and stained by 0.1% (w/v) crystal violet. Each sample was run in triplicate and repeated multiple times.

### Wound-healing migration assay

Cells were seeded into 35-mm plates until they were confluent, and the plates were scraped using a sterile pipette tip to create a wound through the confluent monolayer, then cultured in serum-free medium for 12 hours and photographed at 0 and 12 hours, respectively. The distance of wound migration was measured for further analyses.

### Luciferase assay

193bp fragment of human MMP9 3' UTR with wild or mutant miRNA-responsive elements was cloned into psiCHECK2 vector (Promaga, USA) downstream of the Renilla luciferase ORF. Cells were plated in 24-well plates and transfected the cDNA with Lipofectamine (Invitrogen) as the manufacturer's instruction. Dual-Luciferase Assay (Promega) was used to calculate luciferase activity according to the manufacturer's manual after 48 hours.

### *In vivo* studies

24 6-8 weeks old nude mice were purchased from NCI and divided into 4 groups: 1×10^6^ SK-HEP-1-Luc transduced with pLKO.1-vector co-injected with matrigel; SK-HEP-1-Luc transduced with pLKO.1-vector co-injected with THP-1; SK-HEP-1-Luc transduced with oemiR-149-5p co-injected with matrigel; SK-HEP-1-Luc transduced with oemiR-149-5p co-injected with THP-1, all the cells were injected into left lobes of liver capsule. IVIS was used to measure tumor growth once a week. The study was carried out under the approval of the ethics committee of Xiangya Hospital Central South University (Reference number: 2019020078) and followed the *Interdisciplinary Principles and Guidelines for the Use of Animals in Research, Testing, and Education* by the New York Academy of Sciences, Ad Hoc Animal Research Committee.

### Statistical analysis

All statistical was analyzed by SPSS 19.0 system (SPSS Inc, Chicago, IL). The data values were presented as the mean ± SD. Differences in mean values between two groups were analyzed by two-tailed Student's t test and the mean values of more than two groups were compared with one way ANOVA. p≤0.05 was considered to be statistically significant.

## Results

### M2 macrophages can increase the invasion and migration capacity of HCC cells

**Figure 1:**
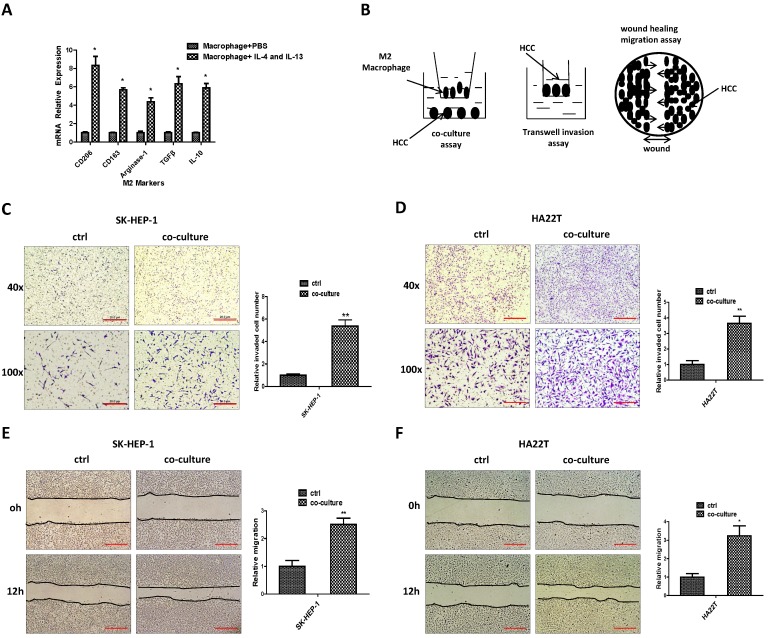
** M2 macrophages can increase the invasion and migration capacity of HCC cells. (A)** qRT-PCR was applied in macrophages using primers for M2 markers (CD206, CD163, Arginase-1,TGFβ, IL-10) after induced by IL-4 and IL-13. **(B)** Pattern diagram for co-culture, invasion and wound healing migration system. **(C-D)** Invasion assay was used to measure invasion capacity of HCC SK-HEP-1 and HA22T cells after co-cultured with M2 macrophages. **(E-F)** Migration capacity was measured in SK-HEP-1 and HA22T cells after co-cultured with M2 macrophages. All quantifications are mean ± SD, *p < 0.05, **p < 0.01.

To study the potential impact of M2 macrophages on HCC progression, we first applied the 100 ng/ml PMA to induce the THP-1 cells into macrophages, then 20 ng/ml IL-4 and IL-13 were used to induce M2 polarized phenotype and the M2 and M1 markers were measured (Fig. 1A, Figure S1 A). We then applied a co-culture system to culture M2 macrophages together with HCC cells for 48hrs (Fig. 1B). Results from invasion assay and “wound-healing” migration assay revealed that the invasion and migration capacity of HCC SK-HEP-1 and HA22T cells were increased after co-culture (Fig.1C-F).

Taken together, results from Fig. 1A-F and Figure S1A suggest that M2 macrophages can increase HCC cells invasion and migration capacity.

### Mechanism dissection of how M2 macrophages increase the HCC cells invasion and migration: via altering the MMP9 expression

**Figure 2:**
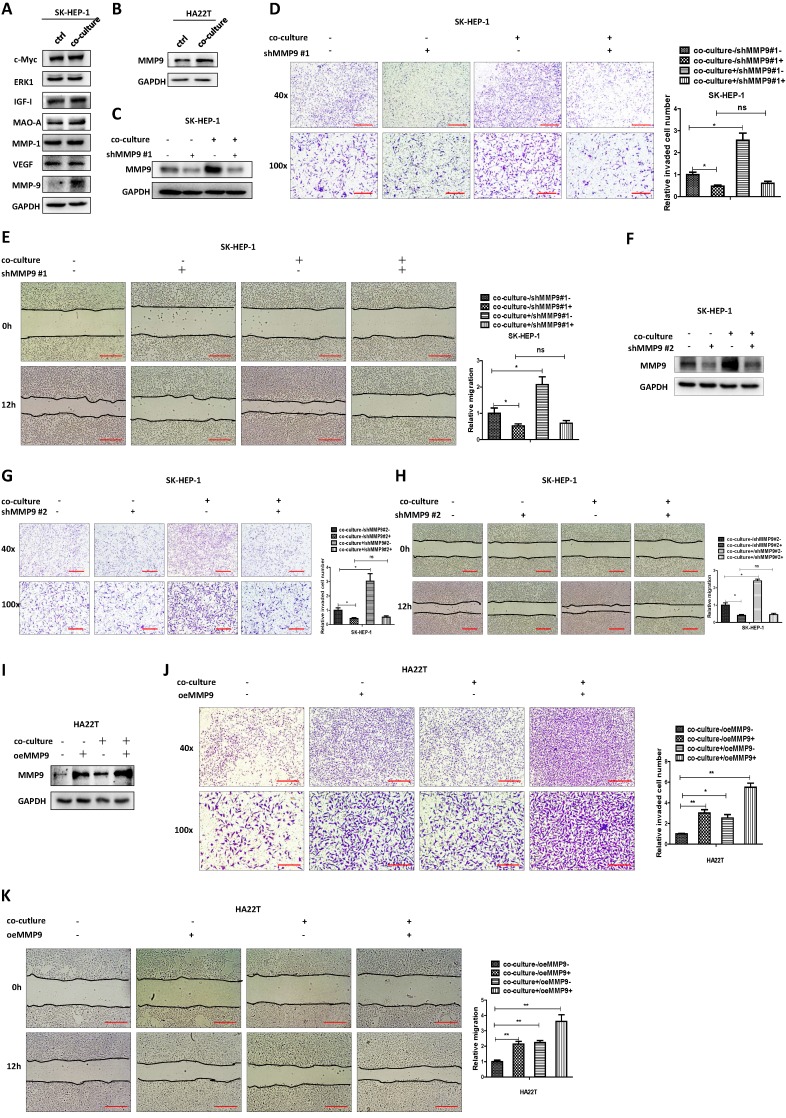
** M2 macrophages increase the HCC cells invasion and migration via altering the MMP9 expression. (A)** Western bolt was used to check related genes expression in SK-HEP-1 cells after co-cultured with M2 macrophages. **(B)** Western blot was used to check MMP9 expression in HA22T cells after co-cultured with M2 macrophages. **(C)** Western blot was used to check MMP9 expression in SK-HEP-1 transfected with MMP9-shRNA#1 in co-culture system. **(D-E)** Invasion and migration capacity were measured in SK-HEP-1 transfected with MMP9 sh-RNA#1 in co-culture system. **(F)** Western blot was used to check MMP9 expression in SK-HEP-1 transfected with MMP9-shRNA#2 in co-culture system. **(G-H)** Invasion and migration capacity were measured in SK-HEP-1 transfected with MMP9 sh-RNA#2 in co-culture system. **(I)** Western blot was used to check MMP9 expression in HA22T overexpressed MMP9 in co-culture system. **(J-K)** Invasion and migration capacity were measured in HA22T overexpressed MMP9 in co-culture system. All quantifications are mean ± SD, *p < 0.05, **p < 0.01, ns: no significant difference.

To examine the detailed mechanisms underlying M2 macrophages' role in regulating HCC cells invasion and migration, we tested the expression of proteins that have been connected with cancer cell invasion and migration 12-18 in both culture alone and in co-culture. The exploratory experiments led to the focus on MMP9 as the results showed that MMP9 protein expression was increased after co-culture, both in SK-HEP-1 and HA22T HCC cells (Fig. 2A-B).

We then examined the potential impact of altered MMP9 on the HCC cell invasion and migration, and results revealed that decreasing MMP9 via adding MMP9-shRNA into SK-HEP-1 cells could reverse M2 macrophages' function to HCC cells (Fig. 2C-E). We also used another shRNA to decrease MMP9 and the results were consistent with the previous data (Fig. 2F-H). In contrast, increasing MMP9 via adding MMP9-cDNA into HA22T cells led to increase of the invasion and migration capacity more significant (Fig. 2I-K).

Together, results from Fig. 2A-K suggest that M2 macrophages may function *via* increasing HCC MMP9 expression to increase the invasion and migration capacity in HCC cells.

### Mechanism dissection of how M2 macrophages increase MMP9 expression of HCC cells: via altering the miR-149-5p

**Figure 3:**
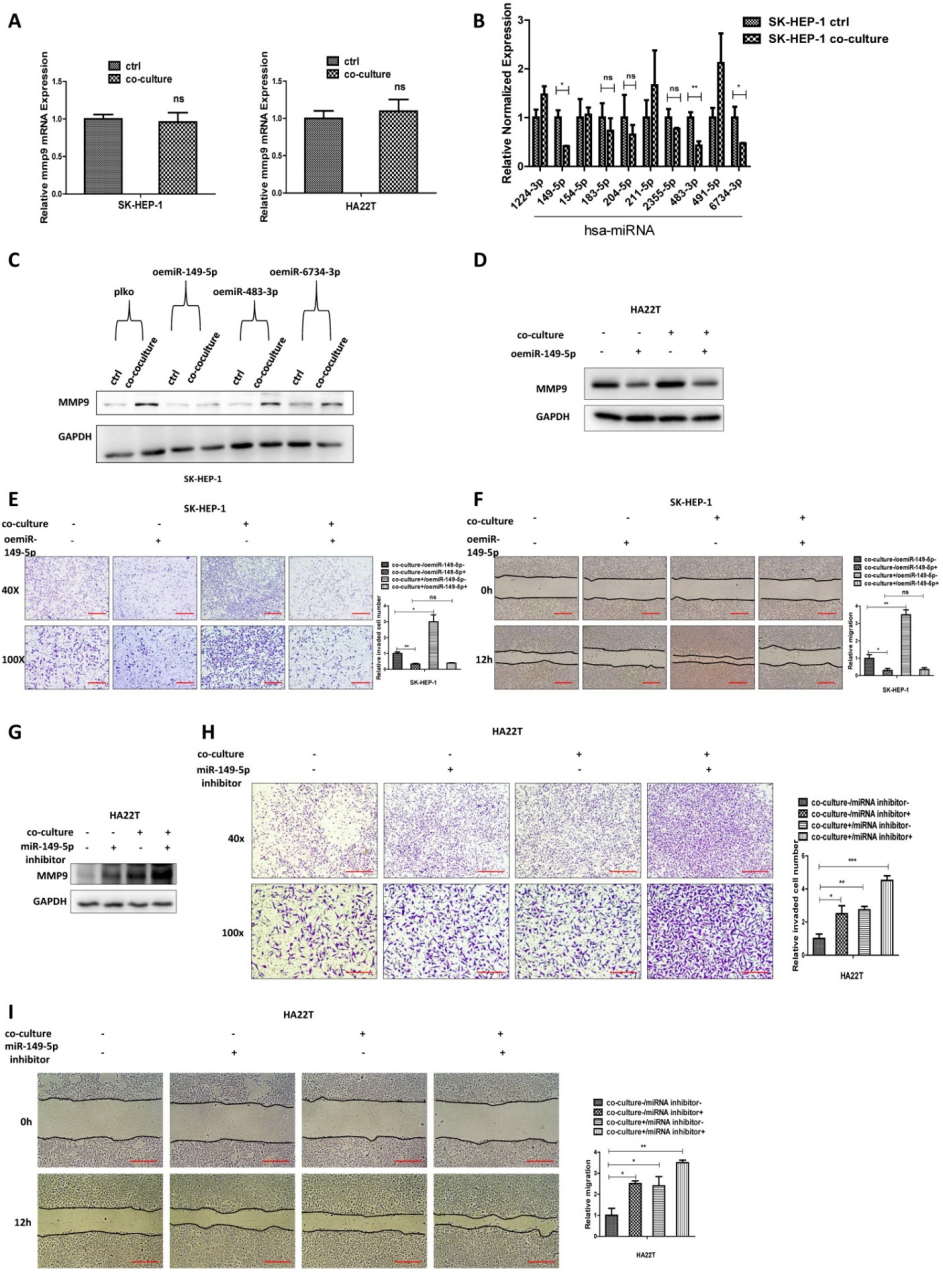
** M2 macrophages increase MMP9 expression of HCC cells via altering the miR-149-5p. (A)** qRT-PCR was used to check MMP9 expression in SK-HEP-1 and HA22T cells after co-cultured with M2 macrophages. **(B)** qRT-PCR was used to check relative miRNAs expression in SK-HEP-1 cells after co-cultured with M2 macrophages. **(C)** Western blot was used to check MMP9 expression after over-expression miR-149-5p/483-3p/6734-3p in SK-HEP-1 cells in co-culture system. **(D)** Western blot was used to check MMP9 expression after over-expression miR-149-5p in HA22T cells in co-culture system. **(E-F)** Invasion and migration capacity were measured after over-expression miR-149-5p in SK-HEP-1 cells in co-culture system. **(G)** Western blot was used to check MMP9 expression after adding miR-149-5p inhibitor in HA22T cells in co-culture system. **(H-I)** Invasion and migration capacity were measured after adding miR-149-5p inhibitor in HA22T cells in co-culture system. All quantifications are mean ± SD, *p < 0.05, **p < 0.01, ***p < 0.001 ns: no significant difference.

To study how M2 macrophages increase HCC MMP9 expression, we first tested the mRNA expression in HCC cells after co-culture and results revealed that the mRNA level of MMP9 didn't increase after co-culture (Fig. 3A).

We also conducted the protein stability assay to check MMP9 protein stability between control and co-culture group, and the result showed no significant difference between these two groups (Figure S1B). These results suggest that M2 macrophages may regulate HCC MMP9 mRNA level through post-transcriptional regulation. So we focus on the miRNAs that can regulate MMP9. We searched the database to select out 10 miRNAs that can regulate MMP9, and then checked miRNAs expression in HCC cells after co-culture; the results revealed that miR149-5p, miR-483-3p and miR-6734-3p were decreased after co-culture (Fig. 3B). Then we over expressed the three miRNAs in HCC cells and co-cultured with M2 macrophages; the results revealed that over expressed miR-149-5p could reverse co-culture's function to MMP9, both in SK-HEP-1 and HA22T cells (Fig. 3C-D), so was the invasion and migration capacity (Fig. 3E-F). On the contrary, when we added miR-149-5p inhibitor in HCC cells, the co-culture's function to MMP9 and invasion/migration became more significant (Fig. 3G-I).

Together, results from Fig. 3A-I and Figure S1B suggest that M2 macrophages may function *via* decreasing miR-149-5p level of HCC cells to increase MMP9 expression.

### Mechanism dissection of how miR-149-5p alters the MMP9 expression under the co-culture condition: via directly target the 3'UTR of MMP9-mRNA

**Figure 4:**
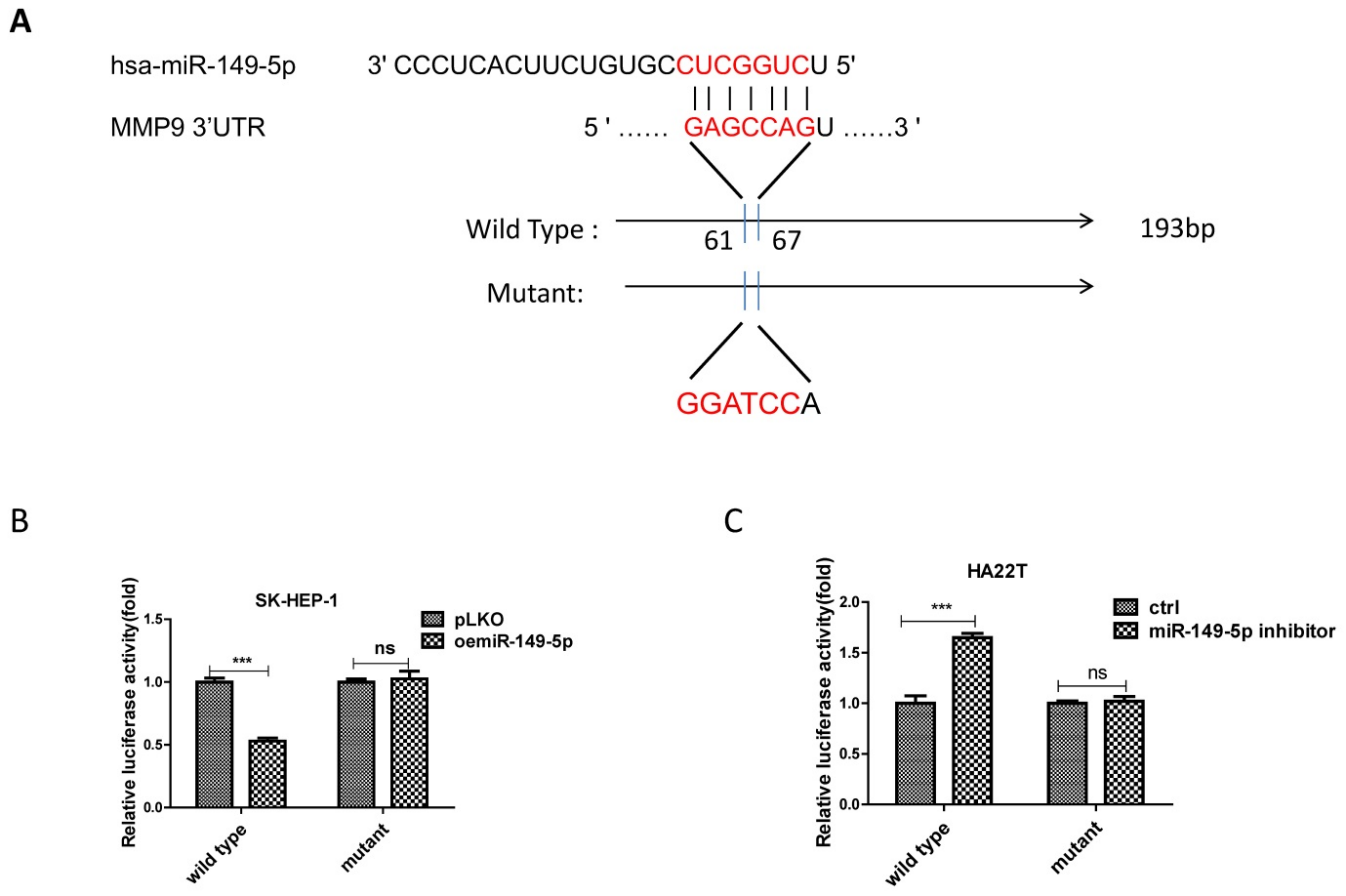
** MiR-149-5p alters the MMP9 expression under the co-culture condition via directly target the 3'UTR of MMP9-mRNA. (A)** Sequence alignment of the MMP9 3'UTR with wild-type versus mutant potential miR-149-5p targeting sites. **(B-C)** Luciferase reporter activity after transfection of wild-type or mutant MMP9 3'UTR reporter construct in SK-HEP-1 and HA22T with over expression miR-149-5p of miR-149-5p inhibitor compared to the control cells.

To dissect the mechanism of how M2 macrophage-miR-149-5p axis can alter the MMP9 expression at the molecular level, we first searched the potential miRNA targeting site located on the 3'UTR of MMP9-mRNA. We then applied the reporter assay with the psiCHECK2 vector carrying the wide-type 3'UTR and a deletion mutant without the miRNA-target site (Fig. 4A). Results from the luciferase assay revealed that addition of miR-149-5p decreased luciferase activity in SK-HEP-1 cells, while addition miR-149-5p inhibitor increased luciferase activity in HA22T cells transfected with wild type MMP9 3'UTR but not the mutant MMP9 3'UTR (Fig. 4B-C), suggesting that miR-149-5p can directly target the 3'UTR of MMP9-mRNA to suppress its protein expression.

### Human Clinical study to link the MMP9 to the HCC progression

**Figure 5:**
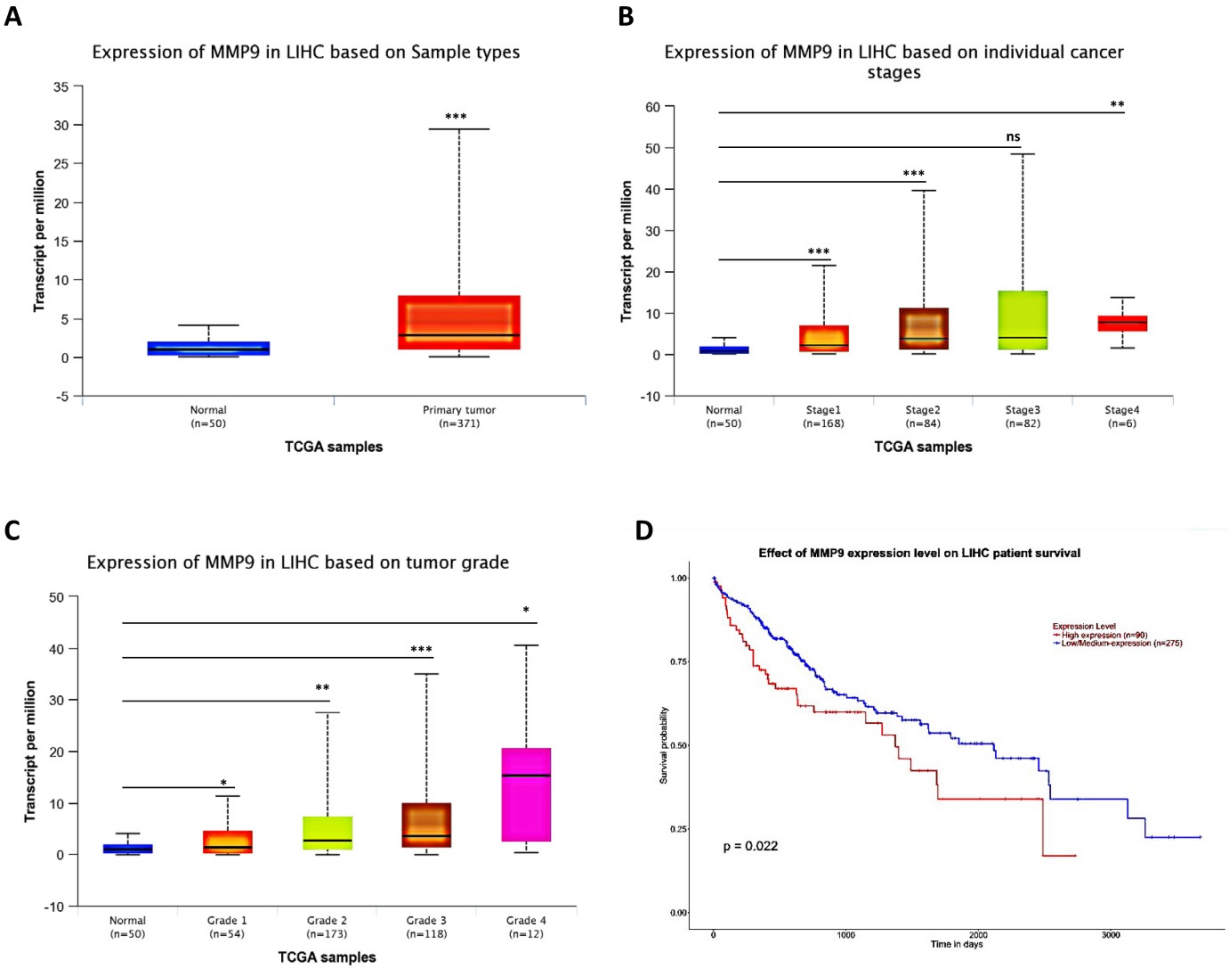
** Human Clinical study to link the miR-149-5p-MMP9 axis to the HCC progression. (A)** HCC patients' from TCGA database shows different expression of MMP9 in normal tissue and primary tumor tissue. **(B)** The TCGA data shows expression of MMP9 in HCC samples based on different individual cancer stages. **(C)** The TCGA data shows expression of MMP9 in HCC samples based on different tumor grade. **(D)** The TCGA data shows effect of MMP9 expression level on HCC patient survival. All quantifications are mean ± SD, *p < 0.05, **p < 0.01, ***p < 0.001 ns: no significant difference.

To link the above in vitro results with human HCC progression, we used UALCAN website (http://ualcan.path.uab.edu/) to analyze cancer transcriptome data in TCGA database. The results showed that MMP9 expression was higher in primary tumor samples than in normal samples (Fig. 5A), and the MMP9 expression level was relatively increased with the increased level of HCC grade and stage (Fig. 5B-C). What's more, the result also showed that high expression level of MMP9 was related to low survival probability (Fig. 5D).

Together, results of clinical data (Fig. 5A-D) from TCGA database proved that MMP9 may play important roles to promote HCC progression.

### Preclinical study using *in vivo* mouse model to prove the roles of M2 macrophages-miR-149-5p-MMP9 axis in the HCC progression

To link our in vitro studies to the clinical significance, we firstly transfected the SK-HEP-1 cells with luciferase reporter gene (for *in vivo* imaging system (IVIS)) to detect tumor progression inside mouse and then transfected the SK-HEP-1-Luc cells with vector control or oemiR-149-5p. The HCC cells were divided into four groups, SK-HEP-1(luc) vector control co-injected with matrigel into the left lobes of liver capsule of nude mouse(Group 1), SK-HEP-1(luc) vector control co-injected with THP-1(Group 2), SK-HEP-1 (luc) oemiR-149-5p co-injected with matrigel (Group 3), SK-HEP-1 (luc) oemiR149-5p co-injected with THP-1 (Group 4). Tumor growth and metastasis were monitored weekly via IVIS analysis.

**Figure 6:**
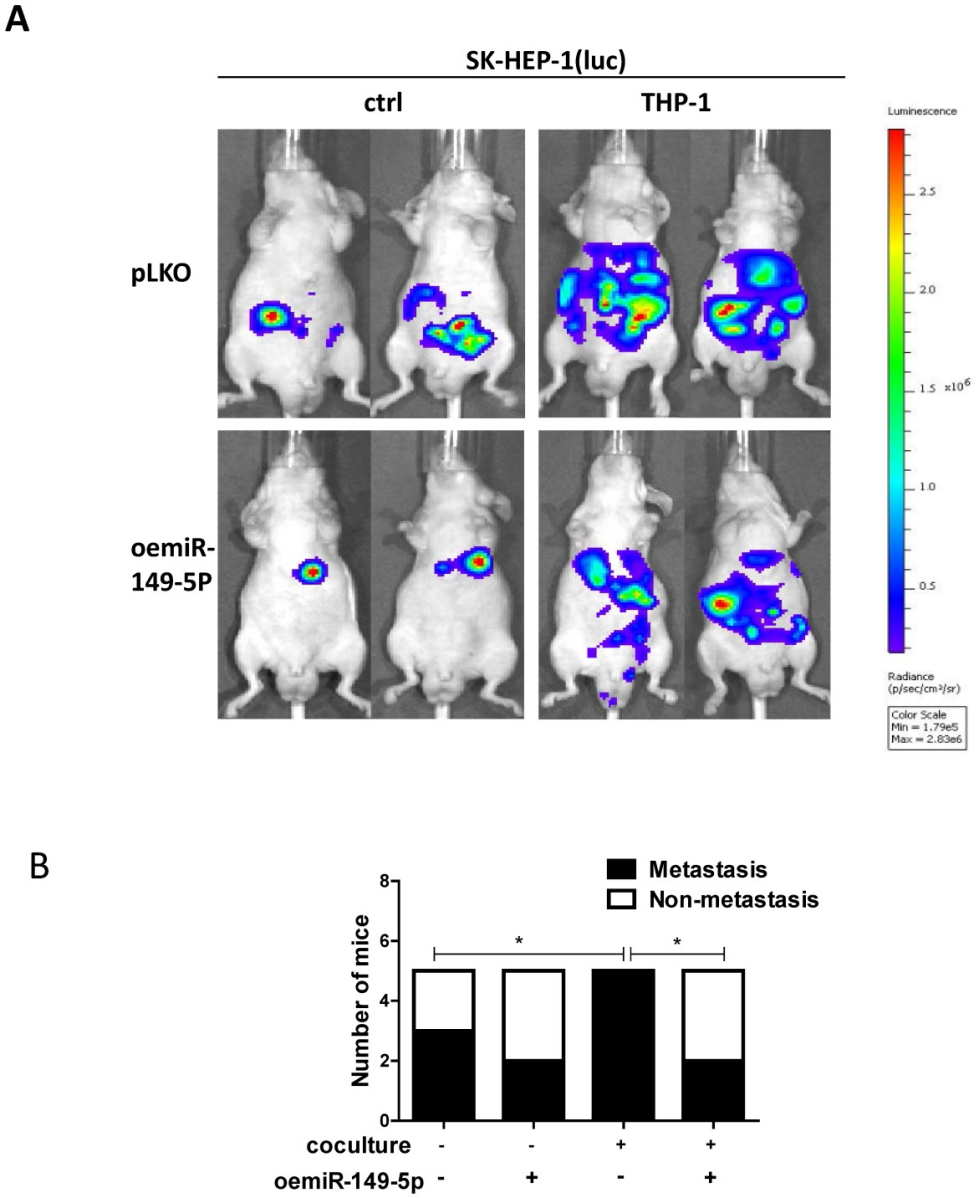
** An *in vivo* mouse model proves the role of M2 macrophage-miR149-5p function in HCC progression. (A)** SK-HEP-1-cells were transduced with Luciferase and with either pLKO or oemiR-149-5p and then co-injected with either matrigel or THP-1 into left lobe of liver and IVIS imaging was used to determine the tumor growth and metastasis in mice in each group. **(B)** Quantitative analysis of number of mice with metastasis in each group. *p < 0.05.

Totally, 20 mice generated tumors (5 for each group), and our results revealed that xenografts of HCC cells with macrophages (THP-1) had more metastasis than no coculture group, and oemiR-149-5p can partly reverse *in vivo* function of macrophages (Fig. 6A-B), and the IHC data of MMP9 expression also confirmed our conclusions(Figure S1C).

Taken together the data from Fig. 6 and Figure S1C, suggests that M2 macrophages/miR-149-5p /MMP9 axis played a critical role to regulate HCC progression, and targeting this newly identified signaling with oemiR-149-5p led to suppression of HCC progression.

## Discussion

HCC is one of the most lethal tumors across the globe. In 2018, 42,220 new cases and 30,200 new deaths of liver and intra hepatic bile duct cancer were counted in the United States 2. High morbidity and mortality make it one of the most burdened tumors in the world. So far, we still lack effective treatments for advanced HCC. Although sorafenib sometimes works, its function is very limited. So, finding new targets and therapies seems to be urgently needed.

More and more evidence proves that the tumor microenvironment is closely related to tumor progression and metastasis, among which, hypoxia and inflammatory cells infiltration such as macrophages seem to be the most important two factors 3-4. M2 macrophage is an alternatively activated phenotype of macrophage and can express anti-inflammatory cytokines to promote tumor progression and metastasis, which is associated with poor outcomes of tumors and has been widely reported in many cancers. Here we provide another example where M2 macrophages can function as a promoter to regulate HCC progression.

MicroRNAs belong to the endogenous small noncoding RNAs family. Usually they play key roles to regulate tumorigenesis and progression by binding to the 3'UTR of target genes mRNA to inhibitor gene expression 19-20. MiR-149-5p is a very important member of microRNA family, which has been widely reported to be a suppressor in tumor progression. For instance, in human osteosarcoma, miR-149-5p can inhibit tumor growth by regulating TWEAK/Fn14/PI3K/AKT pathway 21. For non-small cell lung cancer, the lncRNA-PCAT-1/miR-149-5p/LRIG2 axis plays a key role to regulate its progression 22. Recent study showed that miR-149-5p could inhibit cell proliferation and invasion through targeting GIT1 in medullary thyroid carcinoma 23. All this above proved the key roles of miR-149-5p on tumor progression. In our study, we found miR-149-5p decreased after co-culture, and could increase HCC cells invasion and migration by targeting 3'UTR of MMP9, what's more, by using HCC-oemiR-149-5p cells in *in vitro* and *in vivo* experiment could reverse M2 macrophages' function to HCC cells, which strongly proved miR-149-5p's negative roles on regulating tumor progression.

Matrix metalloproteinases (MMPs) family is a zinc-dependent enzyme which is involved in the degradation of the extracellular matrix both in physiological and pathological processes, such as embryonic development, tissue remodeling, arthritis and tumor progression and metastasis 24. MMP9 is one of key member of MMPs family. Elevating MMP9 expression plays a key role in the progression of many tumors, such as bladder cancer 25, esophageal squamous cell carcinoma 26, and intrahepatic cholangiocarcinoma 27. Our study proved that M2 macrophages can increase HCC cells invasion and migration by upregulating MMP9 expression, this conclusion was strengthened by the outcome in response to the MMP9-shRNA which could block the biochemical and cell behavior induced by M2 macrophages in the HCC tumor microenvironment.

In conclusion, M2 macrophages may increase HCC progression via altering miR-149-5p/MMP9 signaling, and miR-149-5p can target the 3'UTR of MMP9-mRNA to suppress its expression. A potential therapy to target this newly identified signaling may help improve the treatment to better suppress the HCC progression.

## Supplementary Material

Supplementary figures.
