# Supplementary material for: M2 Macrophages Promote HCC Cells Invasion and Migration via miR-149-5p/MMP9 Signaling
Source: J Cancer. 2020 Jan 1;11(5):1277–87. doi: 10.7150/jca.35444 (PMC6959084; doi:10.7150/jca.35444)
Supplement: Supplementary file 1 — Supplementary figures. [file jcav11p1277s1.pdf]

Figure S1

(A) qRT-PCR was applied in macrophages using primers for M1 markers (iNOS and IL-1β) after induced by IL-4 and IL-13. (B) MMP9 protein stability was checked between control and co-culture groups by using CHX(1ug/ml). (C) The IHC for MMP9 of xenografts in four groups. Quantitative analysis is at the right. All quantifications are mean ± SD,$^{*}p < 0.05$, ns: no significant difference.

Figure S2

Schematic model of M2 macrophages promote HCC cells invasion and migration via miR-149-5p/MMP9 signaling.

Figure S1

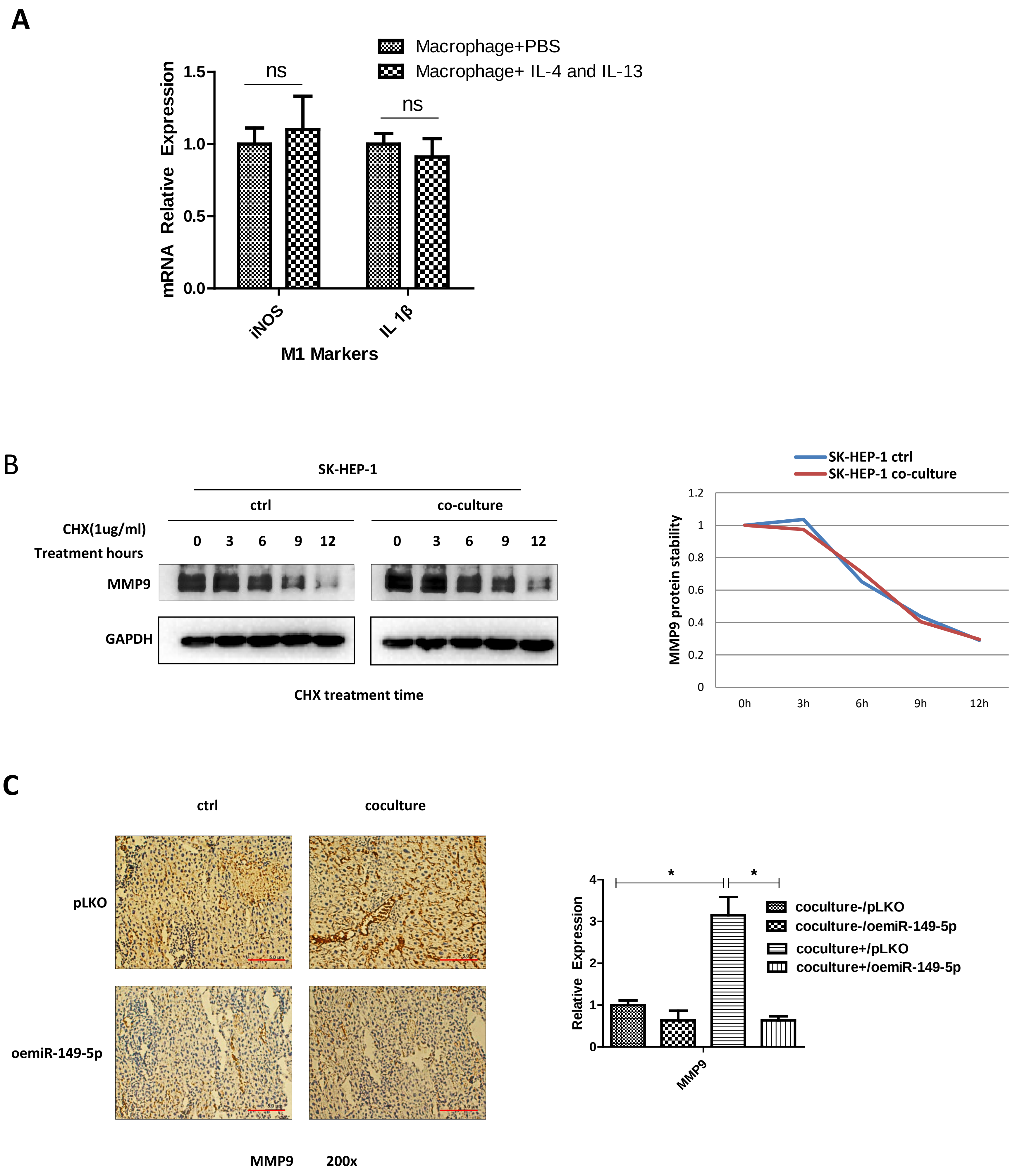
A
Macrophage+PBS
Macrophage+ IL-4 and IL-13
mRNA Relative Expression
1.5
1.0
0.5
0.0
ns
ns
iNOS
IL 1β
M1 Markers
B
SK-HEP-1
ctrl
co-culture
CHX(1ug/ml)
Treatment hours
0 3 6 9 12
0 3 6 9 12
MMP9
GAPDH
CHX treatment time
SK-HEP-1 ctrl
SK-HEP-1 co-culture
MMP9 protein stability
1.2
1
0.8
0.6
0.4
0.2
0
0h 3h 6h 9h 12h
C
ctrl
coculture
pLKO
oemiR-149-5p
MMP9 200x
Relative Expression
4
3
2
1
0
*
*
coculture-/pLKO
coculture-/oemiR-149-5p
coculture+/pLKO
coculture+/oemiR-149-5p
MMP9

Figure S2

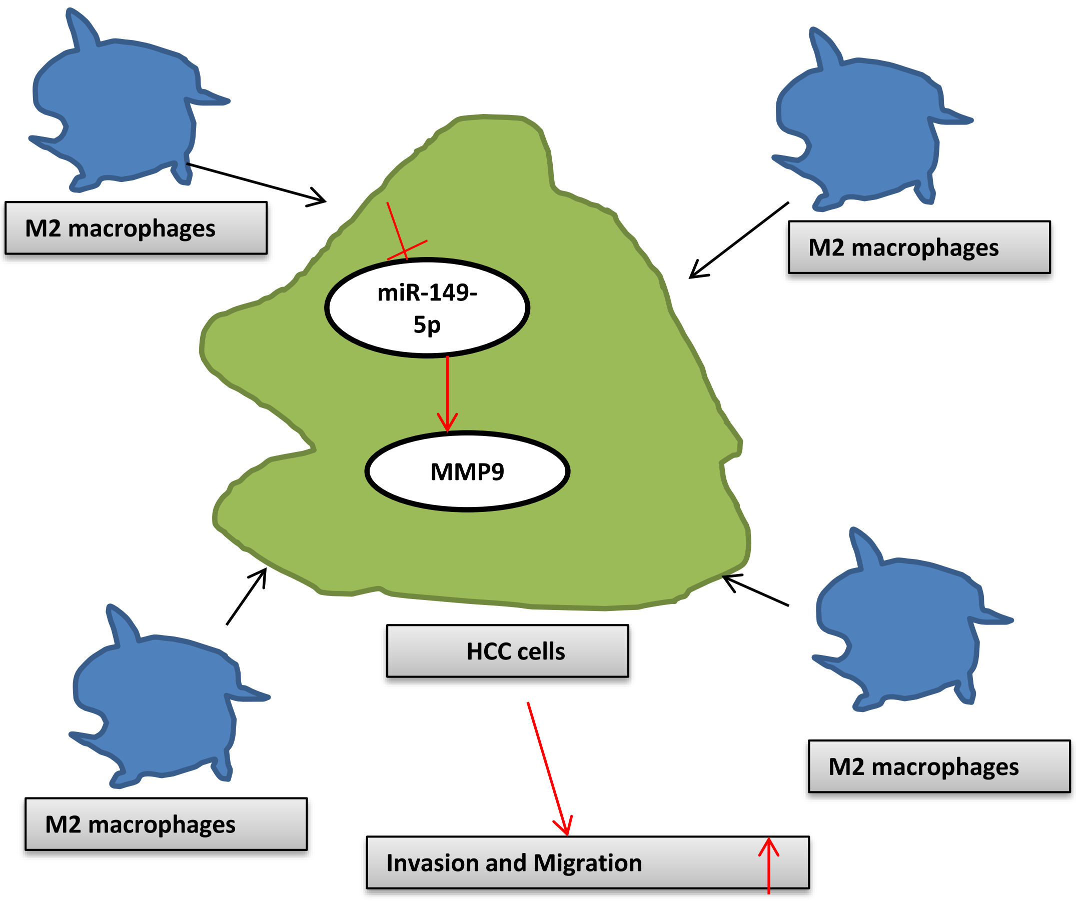
M2 macrophages
M2 macrophages
miR-149-
5p
MMP9
HCC cells
M2 macrophages
M2 macrophages
Invasion and Migration
